# Cultural fit in emotion versus language: a study of Dutch-speaking Belgians and Turkish migrants in Belgium

**DOI:** 10.3389/fpsyg.2024.1488779

**Published:** 2025-01-21

**Authors:** Rüya Su Şencan, Batja Mesquita, Katie Hoemann

**Affiliations:** ^1^Department of Psychology, KU Leuven, Leuven, Belgium; ^2^Department of Psychology, University of Kansas, Lawrence, KS, United States

**Keywords:** culture, emotion, language, emotional fit, cultural fit, immigrant minority

## Abstract

Cultural fit is thought to benefit immigrants’ wellbeing and integration. Previous research on cultural fit focused on explicit attitudes (e.g., how individuals identify with their heritage and host cultures) at the expense of psychological processes (e.g., the extent to which individuals make meaning in similar ways with their surrounding culture). We examined cultural fit in meaning-making in emotional contexts in two complementary ways: first, based on patterns of emotion endorsement (*emotional fit*), second, based on patterns of word use describing emotional situations (*language fit*). Dutch-speaking Belgians and Turkish migrants in Belgium (Ns = 100) described two positive and two negative emotional situations, and rated the intensity of their experience on a set of emotion terms. Language patterns in the descriptions, as quantified by the Linguistic Inquiry and Word Count, distinguished between cultures more effectively than rating patterns. The two fit measures did not converge; they were in fact negatively associated in some analyses, particularly for Turkish migrants’ emotional fit and language fit with Belgian culture, suggesting that when these migrants felt similar emotions, they attended to different aspects of their experience. Future research should disentangle the implications of various types of cultural fit on outcomes relevant to immigrant minorities.

## Introduction

Immigration brings together cultural groups that differ in a variety of ways. Social psychological research on immigration often centers on acculturation: the gradual changes that occur as a result of repeated contact between immigrant minorities and members of the host countries. This research has traditionally focused on explicit attitudes—how individuals report feeling, thinking, and behaving toward their heritage culture and the host culture—as key metrics for assessing acculturation (Berry, 1992; Phinney, 2003; Zagefka and Brown, 2002). However, this approach overlooks the often subconscious changes in culturally rooted psychological processes, such as emotions. Culture (not necessarily defined by national or ethnic boundaries individuals associate with) is a dynamic system of meaning, shaped by the values, norms, and practices, which is constantly reshaped through those who participate in it (Bolis and Schilbach, 2020; Causadias et al., 2018; Markus and Kitayama, 1991). This system influences how its participants interact with the world, shaping their psychological processes in ways that prepare them for the demands of recurring cultural tasks (Kitayama et al., 2009); thus cultural fit in these processes scaffolds individuals’ functioning in a given society (Mesquita et al., 2019; LaFromboise et al., 1993). Exposure to new cultures and new task demands, therefore, could lead to changes in psychological processes such as emotions that are not subsumed under the acculturation of explicit attitudes.

Responding to this concern, studies have increasingly looked toward implicit measures of *cultural fit—*the extent to which individuals feel, think, and behave in similar ways with their surrounding cultural context (De Leersnyder et al., 2011; Güngör et al., 2013). The present paper contributes to this burgeoning line of research by comparing two implicit measures of cultural fit in the domain of emotions, derived, respectively, from emotion intensity ratings and natural language descriptions of recent emotional experiences, in a sample of Turkish immigrants to Belgium and their Dutch-speaking Belgian counterparts. We focus on meaning-making in emotional contexts as emotions represent ways in which people relate to their social environment in line with their stance and goals (Frijda, 1986; Mesquita, 2010), and may thus provide an important perspective on immigrants’ adjustment to the host culture.

## Meaning-making within and across cultures

People vary in their habitual ways of meaning-making, and this is true for emotional situations as well. Take the case of being reprimanded. One person may be sensitive to the presence of others during a reprimand, whereas another person may not be; likewise, the first person may see this reprimand as diminishing social worth, whereas the second may see it as blocking individual goals. One can think of these implicit processes as people’s habits of *attending to* and *evaluating* their experiences of the world (i.e., ways of meaning-making). These patterns of meaning-making are shaped by cultural values, which provide a framework for prioritizing certain goals and interpreting experiences. Cultural values are principles that guide individuals’ priorities (Schwartz et al., 2015), such as fulfilling personal needs (e.g., aligning with individualistic cultural values; Hofstede, 1980) versus establishing social reputation (e.g., aligning with honor values; Leung and Cohen, 2011). Accordingly, these values inform what people attend to in evocative situations and how they evaluate these situations. To the extent that goals, values, and practices differ across cultures, meaning-making (in emotional situations) appears to differ as well (Kitayama et al., 2007; Mesquita et al., 2016; Wu et al., 2021).

In the context of immigration, where individuals must navigate social and psychological landscapes that are unfamiliar, such differences in focus and perspective might create barriers for immigrants to share a common understanding with the members of the local culture, posing challenges for immediate coordination and long-term interpersonal understanding (Higgins, 2016; Mesquita, 2022). On the other hand, previous research on diverse forms of psychological similarity, including similarity in emotional experiences, has shown that cultural fit carries intra- and interpersonal benefits (Consedine et al., 2014; Mobasseri et al., 2019). Studying immigrants’ fit with members of the local culture and how shared understanding is established between them will ultimately grant insight into immigrant minorities’ wellbeing and integration.

## Cultural fit in meaning-making

Extant research has approached cultural fit in meaning-making in two ways. The first examines the emotions that people endorse in response to particular situations (De Leersnyder et al., 2011). Emotion concepts reflect patterns of attention and evaluation guided by a set of values and expectations (Frijda, 1986; Mesquita, 2010). In the examples given above, the first person may feel embarrassed at being reprimanded, reflecting attention to social norms and the evaluation that they have failed to live up to them. The second person may feel angry, focusing on their personal plans and the way these plans have been stymied. The differing emotions of person 1 and 2 reflect different patterns of attention and evaluation, signaling different perspectives. In contrast, having similar emotions in relation to the same situation would have implied that the people involved largely agree about what matters or does not matter, about what should or should not have happened, and what should or should not be done about it (Mesquita et al., 2016; Parkinson, 1996). An alignment of perspective facilitates social connection and supports well-being (Anderson et al., 2003; Gonzaga et al., 2007; Townsend et al., 2014). The same is true for emotional similarity with one’s surrounding cultural context (De Leersnyder et al., 2014, 2015).

A second approach to cultural fit in meaning-making is through natural language. Language serves as a window onto individuals’ subjective experiences; it can be seen as an indicator of mental attention (Boyd and Schwartz, 2021) and psychological processes such as (re)appraisal (Tausczik and Pennebaker, 2010). There is ample evidence demonstrating that when people use similar patterns of language during interactions, they tend to experience more harmonious and fruitful exchanges (Gonzales et al., 2010; Ireland et al., 2011). The fit in language use between an individual and their surrounding cultural context may be similarly impactful. Indeed, a recent study by Srivastava et al. (2018) demonstrated the utility of a language-based approach to cultural fit. Using e-mail communications from a mid-size technology firm, the research team derived patterns of language use with Linguistic Inquiry and Word Count (LIWC; Pennebaker et al., 2007), a software program that counts the percentage of words within a given document that correspond to a set of pre-defined categories ranging from basic linguistic features (e.g., pronouns, verb tenses, negations) to content categories (e.g., social, affective, cognitive), and found that employees’ (dis)similarity with the company norm (i.e., local organizational culture) was associated with outcomes such as retention and exit. The language-based approach to cultural fit used by Srivastava et al. (2018) has yet to be applied to the domain of emotions and tested across national, ethnic, or linguistic boundaries. It is therefore to be seen whether patterns in word use in describing emotional experience distinguish between cultural groups and capture known cultural differences in attention and evaluation, and might thereby have utility in the context of immigration.

## Present study

In the present study, we are interested in cultural fit in emotion and language in the context of emotional situations. The constructs of cultural fit in emotion and cultural fit in language, in principle, reflect overlapping processes of attention and evaluation and both may have positive consequences for people who have transitioned from one cultural context to another. However, the extent to which their measures might be related to each other remains an open question. While endorsing the same patterns of emotions and using the same patterns of words both implicate ways in which people attend to and evaluate the emotional situations, they may be complementary pieces of the emotional experience rather than serving as substitutes for one another. For instance, a person experiencing anger in one situation might focus on the unfair treatment they received, while in another situation, the focus of anger might be on a close relationship history that makes the treatment particularly hurtful. Language can help revealing which aspects (e.g., personal or relational) come to the front in the meaning-making of each situation, in addition to endorsed emotion concepts. These different ways of experiencing an emotion (e.g., anger) are contingent on the situation and might be more typical in one culture than in another (Boiger et al., 2018). Consequently, fit in emotion ratings may, but need not, be associated with language fit.

The present study addressed these open questions by directly comparing cultural fit in emotion based on patterns of emotion endorsement (henceforth, *emotional fit*; De Leersnyder et al., 2011) with cultural fit in language based on patterns of word use (henceforth, *language fit*; Srivastava et al., 2018). By taking into account the co-occurrence of features (i.e., diverse emotion terms, multiple types of words), this approach offers a more comprehensive depiction of the experience than focusing on similarity in single feature (e.g., dysphoria or stress, and use of social or somatic words; Locke and Horowitz, 1990; Townsend et al., 2014; Tsai et al., 2004). The data were collected with Dutch-speaking (majority culture) Belgians and first-generation Turkish migrants in Belgium. Turkish people constitute a substantial portion of the immigrant population in Belgium and there are well-documented differences in interpersonal perceptions, behaviors, and emotional experiences between Turkish and Western European (e.g., Belgian) cultures (Mesquita, 2001; Rodriguez Mosquera et al., 2008; Uskul and Cross, 2019), providing a socially as well as psychologically valuable basis for assessing cultural fit in emotional contexts across these two groups.

In jointly assessing these two approaches to cultural fit, we first tested whether Belgian and Turkish participants evidenced distinct patterns of emotion endorsement and word use when describing emotional situations (Aim 1). We expected to find within-culture and between-culture differences in both fit measures, such that: (H1a) Turkish migrants’ fit with Turkish culture will be higher than their fit with Belgian culture; (H1b) Belgians’ fit with Belgian culture will be higher than their fit with Turkish culture; (H1c) Turkish migrants will fit with Turkish culture more than their Belgian counterparts; (H1d) Belgians will fit with Belgian culture more than their Turkish counterparts. Next, we tested the association between emotional fit and language fit within cultures and in the context of Turkish migrants’ fit with Belgian culture (Aim 2). We predicted that: (H2a) Belgians’ measures of fit with Belgian culture will be positively correlated; (H2b) Turkish migrants’ measures of fit with Turkish culture will be positively correlated; (H2c) Turkish migrants’ measures of fit with Belgian culture will be positively correlated.

## Method

The data analyzed in the present study were collected in the first phase of a large-scale, longitudinal study investigating the role of emotion in the social integration and well-being of immigrant minorities. As detailed below, participants were members of the Belgian (Dutch-speaking) majority in Flanders and recent Turkish migrants to Belgium. The study protocol was reviewed and approved by the KU Leuven Social and Society Ethics Committee. Below we report aspects of the study relevant for the present analyses. All questionnaires administered during data collection, including those that were not used in the present study, are available on https://osf.io/jb74g/?view_only=f9cf9a9a466e4d558d9734197dbd9758.

### Participants

Participants were 100 Dutch-speaking Belgians (53 female, aged 18 to 71, *M_age_* = 38.61, *SD* = 14.80) and 101 Turkish migrants (48 female, aged 19 to 52, *M_age_* = 29.87, *SD* = 6.59) who completed both the initial survey and follow-up interview described below. Turkish migrants were randomly selected from a larger set of 280 Turkish participants for sample size comparability across cultural groups.^1^ A sensitivity analysis conducted in G*Power (Faul et al., 2007) showed that a sample size of 100 provided us with 0.80 power for ɑ < 0.05, two-tailed testing of a bivariate correlation to detect an effect size of *r* ≥ 0.27.

Turkish migrants arrived in Belgium an average of 19.90 months prior to data collection (range 3–53, *SD* = 12.91). Both samples were highly educated: 80 Belgian and 89 Turkish participants held a university or a higher educational degree, 19 Belgian and 10 Turkish participants were high school graduates. Turkish participants reported various reasons for migration, the most frequent of which were living in a safe place (*n* = 54), seeking a new life experience (*n* = 40), studying (*n* = 32), and being a political refugee (*n* = 32).

### Procedure and materials

Participants were recruited from communities in Flanders via personal networks and other convenience sampling strategies (e.g., distributing flyers, contacting organizations). To be eligible, participants needed to be over 18 years old. Sample-specific requirements were as follows: for the Turkish sample, to be directly migrated from Turkey to Belgium between 2 months and 5 years prior to data collection; for the Belgian sample, to have no migration history dating back two generations and to have Dutch as their native language (or in case of French-Dutch bilinguals, to have been speaking Dutch since childhood).

After confirming they met the eligibility criteria, Belgian participants received a link to the online survey to complete on their own. Based on the experience with newcomers in previous studies (e.g., De Leersnyder et al., 2011; Mesquita, 2001), Turkish newcomers were invited to complete the survey in the presence of a research assistant from their cultural background who read the questions out loud and recorded the responses that were orally communicated. Data were collected in two phases: an initial survey, followed by a semi-structured interview. Participants received up to 50€ for completing both parts of the study (20€ for the interview for both groups; 15€ and 30€ for the survey for the Belgian and the Turkish samples, respectively, due to logistic differences explained above).

All participants provided informed consent and took the study including all the materials in their native language. All materials were translated from English in the respective language (Dutch and Turkish) of the participant. Dutch materials were translated by the native Dutch-speakers in the research team. Turkish materials were first translated by a translation company. These translations were back-translated by two independent research assistants who were native speakers of the respective languages. Discrepancies were resolved in group discussions.

#### Initial survey (Emotional Patterns Questionnaire)

As part of this initial survey, all participants completed the Emotional Patterns Questionnaire (EPQ; De Leersnyder et al., 2011). The EPQ consists of prompts asking participants to recall and briefly describe four emotional situations (i.e., events, moments) from the past 6 months. For the Turkish sample, these situations were required to have occurred after their first arrival in Belgium. The elicited situations differ in experienced pleasantness (i.e., valence; positive vs. negative) and interpersonal motive (i.e., motive; relationship-promoting vs. autonomy-promoting), two dimensions that have been found to structure emotional experience across cultures (De Leersnyder et al., 2015; Kitayama et al., 2006). The four situational prompts can be found in Supplementary Table S1.

All participants were asked to report all four situations; however, one Turkish and one Belgian participant failed to report a negative autonomy-promoting situation, and an additional Belgian participant did not report the two relationship-promoting situations. Therefore, we collected 200 positive relationship-promoting (101 Turkish, 99 Belgian), 200 negative relationship-promoting (101 Turkish, 99 Belgian), 201 positive autonomy-promoting (101 Turkish, 100 Belgian), and 199 negative autonomy-promoting (100 Turkish, 99 Belgian) situation descriptions.

After describing each situation, participants were asked to rate the intensity of their emotions at the time of the event on 20 emotion terms using a 5-point Likert scale (1 = *Not at all*; 5 = *Very strongly*). The emotion terms covered a wide range of emotions representing the four emotion types, or combinations of valence and motive (positive relationship-promoting: *respectful toward the others, close to the others, helpful toward the others*; positive autonomy-promoting: *proud, happy, elated [excited]*; negative relationship-promoting: *guilty, ashamed, indebted*; negative autonomy-promoting: *frustrated, angry, resentful*), complemented by emotion terms that are frequently used in literature (*good, bad, calm, worried, nervous, fearful, sad, surprised*). The full scale can be found in the Supplementary Appendix.

#### Semi-structured interviews

After completing the survey, participants consented to take part in an additional interview, in which they described the four EPQ situations in greater detail. The interviews were guided by a set of questions about specific aspects of the situation, such as the time and the place, other people involved, subjective feelings, bodily sensations, and what they and others did. These aspects are commonly cited as core features or components of emotion (e.g., Frijda, 1986), and were intended to help participants to relive and actively evaluate their experience. The interview scheme can be found in the Supplementary Appendix.

Interview duration ranged between 30 and 60 min. Although the original plan was face-to-face data collection, due to COVID-19 regulations the majority of interviews were held online and recorded via Microsoft Teams or Zoom. All interviews were conducted and transcribed by research assistants fluent in the respective languages.

### Data preparation

#### Prompt check

We checked whether participants recalled situations that corresponded with the prompts in two ways. We started by checking, in a two-step process, whether the reported situations met the valence of the prompt. In the first step, two independent coders (native speakers of the respective languages) read the open-ended descriptions of the emotional situations and made a three-level assessment on valence (positive, negative, unclear). Both disagreements and the descriptions evaluated as unclear were discussed among the two coders and a final decision was made as to whether the situation was positive, negative, or unclear. As a second step, we checked whether the ratings of the situation as *good* and *bad* corresponded with the valence prompts. We considered a situation as matching when at least one criterion (either the coders’ assessment, or the rating as good or bad) was clearly met. Therefore, even if the situation met the valence of the prompt on one criterion, and was neutral on the other (i.e., a description coded as unclear or equal ratings of *good* and *bad*), we considered the prompt met. However, when one criterion contradicted the expected valence, we excluded the data from the analyses concerning that situation type. When both of these criteria were neutral, we checked the ratings for *happy* and *angry*, under the expectation that *happy* (*angry*) ratings would be higher for positive (negative) situations. Based on these valence-based checks, data were excluded for 9 situations^2^ in the Turkish sample, and 12 situations^3^ in the Belgian sample.

Next, we confirmed that participants recalled situations that corresponded with the prompts in terms of both interpersonal motive and valence, using the intensity ratings. For each culture, we calculated the average ratings on the four emotion types (positive relationship-promoting, positive autonomy-promoting, negative relationship-promoting, negative autonomy-promoting) and compared them across situations using repeated measures ANOVAs. Post-hoc comparisons with Bonferroni corrections revealed that in all situations, and for both Belgian and Turkish participants, the ratings for the emotion type matching the prompt were higher than the ratings for the other emotion types (see Supplementary Table S2). Therefore, both the manual examination of the content and the analysis of the corresponding intensity ratings confirmed that participants followed the prompts well.

#### Cultural equivalence

We checked for measurement equivalence in the emotion terms across the two cultural groups using Simultaneous Component Analysis (SCA; De Roover et al., 2012). This method allowed us to examine the structural similarities of multiple variables (i.e., emotion terms) across multiple participant blocks (i.e., cultural groups). We conducted SCA on the emotion ratings (18 terms, excluding *good* and *bad* that were only included for valence check purposes) from all four situations, with two blocks (Turkish, Belgian), and allowing for five components (one up to four expected components; i.e., emotion types based on valence and interpersonal motive). A three-component solution was the best fit to the data for both groups, explaining 64.17% of the total variance. Based on this solution, we decided to exclude three emotion terms from further analyses. Two of these terms (*frustrated* and *calm*) loaded on different components across cultures. The third term (*surprised*) loaded on a component that included negatively valenced, autonomy-promoting emotions (e.g., *anger*). This was unexpected given that *surprised* has previously loaded together with positive autonomy-promoting emotions (e.g., *proud*; e.g., De Leersnyder et al., 2011, Study 2), or has loaded differently across cultural groups (e.g., De Leersnyder et al., 2011, Study 1). With comparability in mind, we decided to exclude *surprised* from the present calculations, leaving us with 15 emotion terms (*respectful toward the others, close to the others, helpful toward the others*, *proud, happy, elated [excited]*, *guilty, ashamed, indebted*, *angry, resentful, worried, nervous, fearful, sad*).

#### Emotional fit

We used the emotion intensity ratings from the EPQ to calculate the extent to which individuals’ patterns of emotional experience fit with the average patterns of the respective culture in a situation type. As detailed further below, we analyzed the data separately for each of the four situation types (positive relationship-promoting, positive autonomy-promoting, negative relationship-promoting, and negative autonomy-promoting).

Emotional fit was calculated using profile correlations, following De Leersnyder et al. (2011; see Supplementary Figure S1 for an illustration). First, we created profiles of the 15 emotion intensity ratings for each participant, separately for each of the four situations. Then, for each situation type, we calculated the average emotional patterns of the two cultural groups by averaging all the participants’ profiles in the respective cultural sample. Lastly, we correlated each participant’s profile with (1) the average Belgian pattern obtained from the majority Belgian sample and (2) the average Turkish pattern obtained from the immigrant Turkish sample. When calculating fit within culture (e.g., fit of Belgians to the average Belgian pattern), the individual’s own profile was excluded from the average cultural profile to avoid inflating concordance. The resulting Pearson correlation coefficients were fisher-transformed to be used as estimates of *emotional fit*, representing the similarity of an individual’s emotional pattern to the average emotional pattern in that culture within the given situation type. We calculated four types of emotional fit in this way: Belgian majorities’ fit to the Belgian pattern, Turkish migrants’ fit to the Turkish pattern, Turkish migrants’ fit to the Belgian pattern, and Belgian majorities’ fit to the Turkish pattern.

#### Language fit

We used the transcripts from the semi-structured interviews to calculate the extent to which the patterns of language people used to describe the elicited situations fit with those used by the respective culture. We began by manually splitting the transcripts by situation type (four documents per participant), and then used freely-available software (ConverSplitterPlus, 2022) to generate documents with only participant (and no interviewer) speech.

Transcripts were automatically translated into English using DeepL translate (Dutch: March, 2022; Turkish: September, 2023), for two reasons. First, comparing the results of word counting analyses in two languages introduces a measurement variance problem, as it effectively involves the use of two measurement tools (e.g., one for Turkish and another for Dutch). Any difference observed could be an artifact of the tools rather than an actual difference in the construct of interest. Second, comparing certain features of language (e.g., the use of pronouns) is challenged by inherent differences in the structure of Dutch and Turkish (e.g., Turkish is a pro-drop language, allowing for the omission of explicit subject pronouns due to the verb inflections that convey this information). Translating the documents into English avoided these issues, ensuring that language features were measured in a comparable manner, and aligned with recent recommendations about cross-linguistic comparisons with LIWC (Boyd et al., 2022). Nevertheless, we also conducted analyses using the original languages. These did not change the overall conclusions we draw from the results and are reported in the Supplementary materials (pp. 2–4 and Supplementary Tables S7–S9).

Patterns of language use were quantified using LIWC. We used the 2007 version of LIWC (Pennebaker et al., 2007) for ease of comparison with the original language analyses, as only the 2007 version of LIWC has been translated into Turkish. LIWC2007 includes over 60 categories organized in a structure of parent (e.g., *Social processes*) and sub-categories (e.g., *Family*, *Friends*, *Humans*). We submitted the translated situation-level, participant-only documents to the English LIWC2007. To ensure that Dutch and Turkish interviews are covered by the LIWC to similar extents, we compared the dictionary coverage across the two samples using an independent samples *t*-test, which showed no difference, *t*(801) = 0.02, *p* = 0.99. The English LIWC captured 95.26% of the words used in both of the translated Dutch and Turkish transcripts.

To calculate cultural fit in language, we followed the strategy we used with the emotional fit, this time using LIWC category scores to create profiles. Following Srivastava et al. (2018), we included all LIWC categories except for punctuations, as these were at the discretion of the transcriber. This provided us with 64 word categories (see Supplementary Table S3 for the complete list). Using these categories, we created a profile for each participant for each situation and then averaged these profiles within each culture to obtain the cultural pattern of language use in the respective situation. We correlated each participant’s profile with the average cultural profile (again, the individual’s own profile was excluded from the average cultural profile when fit to one’s own culture was calculated) and Fisher-transformed the correlation coefficients to obtain *language fit*, representing the similarity of an individual’s language pattern to the average language pattern in that culture. As with emotional fit, language fit was computed at the situation level.

We also calculated language fit in an alternative way, using only categories under Psychological Processes (Pennebaker et al., 2007) that are thought to be most relevant for describing emotional experience (i.e., affective processes, social processes, cognitive processes, perceptual processes, biological processes, and their sub-categories; 25 categories in total). This allowed us not only to zoom into the “content” of emotional experience and the relative emphasis given to different aspects of it, for a more focused test of association between language fit and emotional fit in the domain of emotions (Aim 2), but also to ensure that our results are not heavily influenced by function words that might make the comparison in English questionable (e.g., Turkish does not make use of *articles* and allows speakers to drop explicit pronouns as this information is encoded in the verb). These analyses did not change the overall conclusions we draw from the results and are reported in Supplementary materials (pp. 3–4 and Supplementary Tables S9, S10).

## Results

### Descriptive analyses

As a first step, to give an overview of the types of cultural differences, we examined differences in emotion intensity ratings and in word use at the individual level (aggregated across situations) using independent samples *t*-tests. Below we highlight example differences of note. Full results of these analyses can be seen in Supplementary Tables S4, S5.

As for the patterns of emotion endorsement, Turkish migrants, overall, reported higher intensities compared to majority Belgians, including for feeling *respectful* (*t*(192) = 6.27, *p* < 0.001, 95% [0.429; 0.822]), *ashamed* (*t*(192) = 2.57, *p* = 0.01, 95% CI [0.047; 0.357]), and *nervous* (*t*(192) = 4.77, *p* < 0.001, 95% CI [0.3; 0.723]). Belgian majority members did not endorse any emotion with a higher intensity than Turkish migrants.

As for the patterns of language use, Turkish migrants used proportionately more words than majority Belgians for third-person singular pronouns (e.g., *she*, *oneself; t*(198) = 3.54, *p* < 0.001, 95% CI [0.185; 0.648]) and social processes (e.g., *mate*, *talk*; *t*(198) = 5.92, *p* < 0.001, 95% CI [1.119; 2.237]), and also for personal concerns in the professional domain, such as achievement (e.g., *earn*, *win*; *t*(198) = 4.69, *p* < 0.001, 95% CI [−0.659; −0.177]). Belgians, on the other hand, used proportionately more words for second-person singular pronouns (e.g., *you*, *yours*; *t*(198) = − 3.41, *p* < 0.001, 95% CI [0.183; 0.448]) and some of the cognitive processes, such as certainty (e.g., *absolute*, *never*; *t*(198) = −8.23, *p* < 0.001, 95% CI [−0.469; −0.288]). Although the two cultural samples did not differ in their proportional use of words related to overall affective processes and more particularly positive affect, Turkish migrants used proportionately more words related to negative affect than Belgians (e.g., *hurt*, *ugly*; *t*(198) = 2.45, *p* = 0.02, 95% CI [0.03; 0.279]).

### Confirmatory analyses

Before testing our hypotheses, we checked if we could create composite estimates of emotional fit and language fit by averaging the situation-level coefficients for each measure. Reliability analyses of fit estimates across the four situation types showed poor reliability for emotional fit, with Cronbach’s *α* of 0.25 for fit with the Turkish culture (0.20 in the Turkish migrant sample; 0.22 in the Belgian majority sample) and of 0.17 for fit with the Belgian culture (0.15 in the Turkish migrant sample; 0.22 in the Belgian majority sample). Cronbach’s alpha for language fit with the Turkish culture was 0.79 (0.61 in the Turkish migrant sample; 0.70 in the Belgian majority sample), and for language fit with Belgian culture was 0.86 (0.68 in both cultural samples). Although reliabilities for language fit were acceptable, we conducted all analyses at the situation level to keep our analyses and interpretations comparable across the two fit measures.

#### Aim 1: Do Belgian and Turkish participants evidenced distinct patterns of emotion endorsement and word use?

To meet our first aim, that is to answer whether Belgian and Turkish participants evidenced distinct patterns of emotion endorsement and word use, we conducted within- and between-culture comparisons of emotional fit and language fit across the four situation types. All comparisons can be seen in Table 1.

**Table 1:** Within- and between-culture comparisons in measures of cultural fit.

Emotional fit				
Within culture comparisons				
Situation type	Cultural group	Mean (SD)		t (df)
		Fit to Turkish	Fit to Belgian	
Positive Relationship-promoting	Turkish	1.35 (0.54)	1.33 (0.53)	3.11** (100)
	Belgian	1.32 (0.52)	1.35 (0.55)	2.41* (96)
Positive Autonomy-promoting	Turkish	1.41 (0.53)	1.40 (0.52)	1.80 (99)
	Belgian	1.29 (0.55)	1.29 (0.55)	0.04 (96)
Negative Relationship-promoting	Turkish	0.75 (0.38)	0.65 (0.46)	4.02*** (95)
	Belgian	0.66 (0.26)	0.81 (0.35)	6.24*** (93)
Negative Autonomy-promoting	Turkish	0.94 (0.41)	0.83 (0.38)	5.72*** (96)
	Belgian	0.74 (0.31)	0.84 (0.35)	5.57*** (95)

Between culture comparisons			
Situation type	Fit type	Turkish–Belgian (SE)	t (df)
Positive Relationship-promoting	Fit to Turkish	0.03 (0.08)	0.41 (196)
	Fit to Belgian	−0.03 (0.08)	−0.33 (196)
Positive Autonomy-promoting	Fit to Turkish	0.12 (0.08)	1.54 (195)
	Fit to Belgian	0.11 (0.08)	1.40 (195)
Negative Relationship-promoting	Fit to Turkish	0.09 (0.05)	1.81 (188)
	Fit to Belgian	−0.16 (0.06)	−2.64** (188)
Negative Autonomy-promoting	Fit to Turkish	0.19 (0.05)	3.66*** (191)
	Fit to Belgian	−0.01 (0.05)	−0.18 (191)

Language fit				
Within culture comparisons				
Situation type	Cultural group	Mean (SD)		t (df)
		Fit to Turkish	Fit to Belgian	
Positive Relationship-promoting	Turkish	2.77 (0.24)	2.46 (0.18)	18.27*** (99)
	Belgian	2.47 (0.18)	2.79 (0.24)	19.02*** (96)
Positive Autonomy-promoting	Turkish	2.67 (0.26)	2.43 (0.18)	15.54*** (99)
	Belgian	2.49 (0.18)	2.77 (0.22)	17.40*** (96)
Negative Relationship-promoting	Turkish	2.82 (0.24)	2.51 (0.18)	17.86*** (95)
	Belgian	2.53 (0.21)	2.86 (0.26)	18.35*** (94)
Negative Autonomy-promoting	Turkish	2.84 (0.27)	2.52 (0.20)	15.47*** (96)
	Belgian	2.55 (0.22)	2.89 (0.26)	17.36*** (95)

Between culture comparisons			
Situation type	Fit type	Turkish–Belgian (SE)	t (df)
Positive Relationship-promoting	Fit to Turkish	0.30 (0.03)	9.93*** (195)
	Fit to Belgian	−0.33 (0.03)	−11*** (195)
Positive Autonomy-promoting	Fit to Turkish	0.18 (0.03)	5.62*** (195)
	Fit to Belgian	−0.34 (0.03)	−12.03*** (195)
Negative Relationship-promoting	Fit to Turkish	0.29 (0.03)	8.74*** (189)
	Fit to Belgian	−0.35 (0.03)	−10.91*** (189)
Negative Autonomy-promoting	Fit to Turkish	0.30 (0.03)	8.39*** (191)
	Fit to Belgian	−0.37 (0.03)	−11.08*** (191)

All analyses were conducted with two-tailed testing. *p < 0.05, **p < 0.01, ***p < 0.001.

##### Emotional fit

We tested whether people had higher emotional fit with their own cultural group than with the other cultural group using paired-sample *t*-tests. Results offered partial support for H1a and H1b for emotional fit. Turkish migrants indeed fit better with the Turkish culture in three situation types (positive relationship-promoting, negative relationship-promoting, and negative autonomy-promoting; *p*s ≤ 0.002), but not in the positive autonomy-promoting situations (*t*(99) = 1.80, *p* = 0.08). A similar pattern was observed for Belgian majority members, who fit better with the Belgian culture in the same three situations, *p*s ≤ 0.02, but not in positive autonomy-promoting situations (*t*(96) = 0.04, *p* = 0.97).

We also checked whether people had higher emotional fit with their own cultural group than people from the other cultural group using independent samples *t*-tests. Results partially supported H1c and H1d for emotional fit, with the expected patterns emerging only in negative situations and for some cultural comparisons. Specifically, Belgian majority members fit better with Belgian culture (*M* = 0.81, *SD* = 0.35) than Turkish migrants did (*M* = 0.66, *SD* = 0.26) in negative relationship-promoting situations, *t*(188) = 2.64, *p* = 0.009; and Turkish migrants fit better with the Turkish culture (*M* = 0.94, *SD* = 0.41) than Belgians majority members did (*M* = 0.83, *SD* = 0.38) in negative autonomy-promoting situations, *t*(191) = 3.66, *p* < 0.001. There was no evidence for cultural differences in emotional fit in positive situations, *p*s > 0.13.

##### Language fit

Paired-sample *t*-tests for within-culture comparisons revealed that in all four situation types, Turkish migrants’ language fit better with the Turkish culture, *p*s < 0.001, and Belgian majority members’ language fit better with the Belgian culture, *p*s < 0.001. Thus, H1a and H1b were fully supported for language fit.

Independent samples *t*-tests for between-culture comparisons revealed that in all four situation types, Turkish migrants’ language fit better with the Turkish culture than Belgian majority members did, *ps* < 0.001, and Belgian majority members’ language fit better with the Belgian culture than Turkish migrants did, *p*s < 0.001. H1c and H1d were also fully supported for language fit.

##### Exploratory analyses

For both cultural groups and for each measure of cultural fit, we examined the associations between participants’ fit with their own culture and with the other to see whether this would provide insight into why H1a-d were met for language fit but only partially met for emotional fit. The reasoning is that the higher the association between fit with Belgian culture and fit with Turkish culture, the less likely it is that the fit measure captures cultural differences.

For emotional fit, Pearson’s correlations showed that fit with one culture was strongly associated with fit with the other (*rs* ranging from 0.76 to 0.99; see Supplementary Table S6), such that when people fit well with Turkish culture it is highly likely that they also fit well with Belgian culture (and vice versa). A similar pattern was observed for language fit, but to a lesser extent, such that fit with Turkish culture and fit with Belgian culture were positively associated (*rs* ranging from 0.65 to 0.79, see Supplementary Table S6).

Lastly, we tested whether the strength of association between fit with one culture and fit with the other culture statistically differed between emotional fit and language fit. For this purpose, we compared correlation coefficients for the two measures (correlation between emotional fit with Turkish culture and emotional fit with Belgian culture on one hand, and the correlation between language fit with Turkish culture and language fit with Belgian culture on the other hand) within cultures across situations using confidence intervals (Diedenhofen and Musch, 2015; Zou, 2007). Results revealed that, overall, the association between fit with Turkish culture and fit with Belgian culture was higher for emotional fit than for language fit (see Supplementary Table S6).

#### Aim 2: Are emotional fit and language fit associated with each other?

For our second aim, that is to test the association between emotional fit and language fit in emotional situations, we first checked this relationship in Belgian majority and Turkish migrant samples separately. Then, to test the fit approach across cultural boundaries, we examined if Turkish migrants’ language fit with the Belgian culture converged with their emotional fit with the Belgian culture. In all cases, we ran bivariate Pearson’s correlations between language fit and emotional fit using a two-tailed test of significance at *ɑ* = 0.05.

Within-culture analyses showed that emotional fit and language fit were not consistently related to each other in either cultural group. Unexpectedly, Turkish migrants’ language fit and emotional fit with the Belgian culture were negatively associated in negative situations, such that when they reported feelings similar to the average Belgian during unpleasant events, the language features they used to talk about these events differed from those of the average Belgian (*r*(94) = −0.27, *p* = 0.01, 95% CI [−0.446, −0.074] for negative relationship-promoting events; *r*(95) = −0.25, *p* = 0.02, 95% CI [−0.425, −0.049] for negative autonomy-promoting events). There was no relationship between Turkish migrant’s language fit and emotional fit with the Belgian culture in positive situations. Therefore, H2a, H2b, and H2c were not supported. A correlation matrix of the relationship between measures of cultural fit across situations can be seen in Table 2.

**Table 2:** Correlations between language fit and emotional fit.

Situation type	*r*	*p*	*df*
Within-culture: Belgians			
Positive relationship-promoting	0.10	0.35	95
Positive autonomy-promoting	0.08	0.42	95
Negative relationship-promoting	−0.19	0.07	92
Negative autonomy-promoting	0.14	0.18	94
Within-culture: Turkish migrants			
Positive relationship-promoting	−0.08	0.41	99
Positive autonomy-promoting	0.04	0.73	98
Negative relationship-promoting	−0.14	0.18	94
Negative autonomy-promoting	0.02	0.86	95
Between-culture: Turkish migrants’ fit with Belgians			
Positive relationship-promoting	−0.06	0.53	99
Positive autonomy-promoting	−0.06	0.57	98
Negative relationship-promoting	**−0.27**	0.01	94
Negative autonomy-promoting	**−0.25**	0.02	95

The associations that are statistically significant (*p* < 0.05) are represented in bold.

## Discussion

The present study explored Turkish migrants’ cultural fit with Dutch-speaking majority Belgians in the context of emotional situations; with the goal of ultimately better understanding the psychological processes that support adjustment and integration. We employed two implicit measures of cultural fit that are thought to reflect patterns of attention and evaluation in emotional situations. The first, emotional fit, examines the emotions that people endorse in response to four types of emotion-eliciting situations varying in valence (positive vs. negative) and interpersonal motive (relationship- vs. autonomy-promoting; De Leersnyder et al., 2011). The second, language fit, examines the natural language descriptions of these emotional situations with a word counting approach (Srivastava et al., 2018). This study is the first to apply the language fit approach to an immigration context, using verbal descriptions of emotional events as opposed to written e-mail communications used in previous research (Srivastava et al., 2018).

In an initial set of descriptive comparisons, we found that the two cultural groups meaningfully differed both with respect to emotion endorsement and with respect to word use. We then assessed whether there were cultural differences with regard to our two measures of cultural fit –emotional fit and language fit—by comparing each fit measure within and between groups. Consistent with our expectations, we found that language fit clearly differentiated Turkish migrants and Belgian majority members. These cultural differences were weaker and less consistent for emotional fit. Finally, we assessed the relationship between the two measures of cultural fit. Contrary to our expectations, we did not find a positive association between emotional fit and language fit. In fact, in some analyses the association was negative, particularly when we compared Turkish migrants’ ways of fitting with Belgians. An outline of our hypotheses and summary of our findings is presented in Table 3. In what follows, we discuss each set of findings in turn, and conclude by reflecting on relevance for existing theories and considering study limitations and potential future directions.

**Table 3:** Summary of findings.

Aim 1: Do Belgian and Turkish participants evidence distinct patterns of emotion endorsement and word use?		
Hypothesis	Results	Comments
H1a: Turkish migrants’ fit with Turkish culture will be higher than their fit with Belgian culture.	Partially supported for emotional fit (3 of 4 situation types: positive relationship-promoting, negative relationship-promoting, negative autonomy-promoting). Fully supported for language fit.	Exploratory analyses revealed that fit with Belgian culture and fit with Turkish culture was positively correlated, with the correlations being stronger for emotional fit than language fit.
H1b: Belgians’ fit with Belgian culture will be higher than their fit with Turkish culture.	Partially supported for emotional fit (3 of 4 situation types: positive relationship-promoting, negative relationship-promoting, negative autonomy-promoting). Fully supported for language fit.	
H1c: Turkish migrants will fit with Turkish culture more than their Belgian counterparts.	Partially supported for emotional fit (1 of 4 situation types: negative autonomy-promoting). Fully supported for language fit.	
H1d: Belgians will fit with Belgian culture more than their Turkish counterparts.	Partially supported for emotional fit (1 of 4 situation types: negative relationship-promoting). Fully supported for language fit.	

Aim 2: Are Emotional Fit and Language Fit Associated with Each Other?		
Hypothesis	Results	Comments
H2a: Belgians’ measures of fit with Belgian culture will be positively correlated.	Not supported.	
H2b: Turkish migrants’ measures of fit with Turkish culture will be positively correlated.	Not supported.	
H2c: Turkish migrants’ measures of fit with Belgian culture will be positively correlated.	Not supported.	Unexpected negative correlations were found in negative situations.

### Differences in emotion endorsement and word use

Our initial descriptive analyses revealed that Turkish migrants reported higher levels of respect and shame on the emotion scales, along with a greater use of third-person singular (he/she) pronouns and social words than Belgians in the interviews about emotional events. These differences align with prior work suggesting that Turkish and Belgian cultures differ on whether they embody models of individualism or collectivism (Hofstede, 1980) and honor or dignity (Leung and Cohen, 2011). Belgian culture, as part of the broader Western European context, is considered a relatively more individualistic culture where dignity values prevail: autonomy, independence, and achievement of individual goals are prioritized, and self-worth is viewed as intrinsic. Turkish culture is considered a relatively more collectivistic culture that endorses honor values to a greater extent: interdependence, harmony, and group goals are prioritized, and self-worth is determined both internally and externally—based on social status and reputation (Boiger et al., 2013, 2014; Mesquita, 2001; Uskul and Cross, 2019). In the introductory example of a reprimand situation, the first person focused on their esteem in the eyes of others and felt embarrassed, while the second person focused on the obstacles imposed by others on their personal plans and felt angry. These example patterns of attention and evaluation in emotional situations reflect the experiences of prototypical Turkish and Belgian culture members, respectively, and are partially borne out by the current data. Turkish migrants’ emphasis on social aspects of experience, coupled with more intense feelings of respect and shame, is in line with a cultural model where collectivism and honor values are endorsed.

The goal of our initial descriptive analyses was to illustrate how particular emotions are endorsed or categories of words are used by members of the two cultural groups, and how these together might reflect (cultural) patterns of attention and evaluation with regard to emotional situations. To this end, we neither offer an exhaustive comparison of emotion endorsement and word use between the two cultural samples, nor claim that the differences we observed are solely reflective of broader cultural models. Rather, we acknowledge that some of these differences might also be due to sample-specific characteristics. For instance, Turkish migrants’ attention to achievement concerns might be explained by the need to compete for resources in historically rough contexts where external structures for protecting individual rights are weak (consistent with an honor culture model; Leung and Cohen, 2011), or simply by the highly educated nature of our sample, who predominantly migrated to pursue higher education or highly qualified jobs. The ultimate purpose of the present research is not to exhaustively capture, or explain, the cultural similarities and differences between these cultural samples, but rather to illustrate that the current measures yield cultural fit and misfit in meaning-making in a complementary way. Therefore, instead of unpacking all the differences in emotion endorsement and word use, we consider the observations we obtained from these comparisons sufficient to assert that we are capturing meaningful patterns of attention and evaluation in emotional contexts, regardless of whether these reflect broader cultural models or sample-specific characteristics.

### Cultural fit in patterns of emotion endorsement and word use

Our comparisons of each measure of cultural fit within and between groups suggested that language fit may be more reliable than emotional fit in differentiating cultural groups. To follow up on the inconsistent cultural differences we observed in emotional fit (H1a-d), we examined the associations between fitting with one culture and with the other. For both measures of fit, people who fit better with one culture also fit better with the other. However, our results showed that this positive association was stronger for emotional fit than for language fit. This imbalance might partly be explained by methodological differences in calculating the two types of fit. While we included all types of language properties (i.e., all LIWC dictionaries) when estimating language fit, we removed the emotion items that clustered differently across cultures when estimating emotional fit. This preprocessing step is intended to ensure cultural equivalence of the emotion concepts being compared, but leaves out items that cross-culturally vary in meaning. It may thus lead to an underestimation of actual differences in emotional fit—something that does not occur in calculating language fit. Also, emotional fit might be more susceptible to prompt-specific constraints than language fit, because the emotion items used in the rating task were already designed to match with the types of situations requested (e.g., valence) in contrast to most language categories used to estimate language fit (exceptions are positive vs. negative affect categories). Therefore, if people endorsed the anticipated emotions (based on the valence prompt) in a given situation type, cross-cultural differences in patterns of emotion endorsement would be overshadowed.

An alternative explanation for why fit in language use more effectively differentiates cultures than fit in emotion endorsement could be that language people use to describe their experiences is more influenced by the display norms prescribed by their culture (thus reflecting what individuals were at ease to *express*), whereas their emotion endorsement is less affected by these norms (thus more directly reflecting ‘true’ emotional *experience*). While the distinction between emotional expression and experience has often been discussed in explaining cultural differences in emotions (De Leersnyder et al., 2013; Matsumoto et al., 2008), our approach focuses on how language and emotions are both embedded in cultural systems of meaning-making. From this perspective, one is no more “authentic” than the other; in principle, they should each reflect (different aspects of) ‘cultural fit.’ However, our methods also might have contributed to this observed difference: language fit was estimated from semi-structured interviews that allowed participants to focus on the aspects they preferred (possibly in line with their cultural values), whereas emotional fit was estimated from closed-format rating scales, which might have constrained participants’ ability to convey cultural nuances. Future research employing open-ended measures to assess emotional fit, for example by scoring texts for specific emotions (Aroyehun et al., 2023; Mohammad, 2016; Raji and De Melo, 2020), could offer richer insights into how emotions are integrated within cultural meaning-making systems.

The lack of a consistent relationship between emotional fit and language fit (H2a-c) suggests that these measures may capture distinct components of emotional meaning-making. Language fit, as we measured using a word counting approach, might provide more insight into attentional processes—that is, how attention is deployed across a wide range of situational features—than it does into evaluative processes. Indeed, prior work used word counting approaches to language to examine how different features (e.g., bodily sensations, cognitive processes, involvement of others) are foregrounded in descriptions of emotional experiences across cultures. For instance, Tsai et al. (2004) found that Chinese American migrants’ focus on social and somatic aspects varied as a function of their acculturation orientation. Emotion ratings, on the other hand, may give more insight into evaluative processes than word counting approaches do, as emotions are thought to emerge from a comparison between one’s current state and a desired state in line with values and expectations (Frijda, 1986; Mesquita, 2010; Parkinson, 1996). To illustrate, following the example of a reprimand, while our first person was embarrassed because of their attention to social aspects of the situation, and our second person was angry because of goal obstruction, a third person might have felt angry not because their goals were blocked but because their social reputation was damaged; therefore, they would be focused on social aspects of the experience and likely use words with social connotations (like the first person) but they would still endorse anger as they would attribute blame to the reprimander (like the second person); therefore, the two ways of fitting may not always go hand in hand. Alternatively, as discussed above, language fit might be more reflective of what participants preferred to express; or the lack of association could be simply due to the different stages of data collection, as conducting the interview after the initial survey provided participants with the opportunity to re-evaluate their experiences.

The negative association between emotional fit and language fit in certain analyses, particularly regarding Turkish migrants’ fit with majority Belgian culture, is noteworthy. These results suggest that when Turkish migrants reported feeling similarly to an average Belgian, or evaluated the situation similarly, they tended to describe their experience with different sets of words, or attended to different features in their descriptions. The fact that the negative association between emotional fit and language fit particularly emerged in the immigrants’ fit with the majority culture, rather than people’s fit with their own culture, suggests that this divergence may itself be interpreted as a form of cultural misfit. Future research could explore how this form of misfit might predict various outcomes for immigrant minorities, such as psychological well-being and social integration. Taken together with the results showing that language fit more reliably differentiated cultural groups, these findings suggest that looking at how people construct their experiences through language may be a valuable contribution to our understanding of cultural fit in meaning-making in emotional contexts, above and beyond emotional fit.

### Relevance for existing theories

The present study speaks to emotion theories, as well as theories of acculturation. First, by incorporating language into the study of emotion, it extends the conceptualization of emotion beyond traditional rating scale approaches, which often fail to capture aspects relevant to meaning-making. Our research uses the concept of meaning-making in emotional contexts to explore cultural fit. While meaning-making aligns with appraisal—the process by which individuals evaluate emotional situations in relation to their current concerns (Moors et al., 2013)—it goes beyond appraisals by capturing various ways of interacting with the environment and encompassing a broader range of cognitive and attentional processes. For instance, meaning-making involves not only how individuals evaluate their environment (e.g., as unfair), but also which aspects of their experience were foregrounded (e.g., affect vs. cognition, which were captured by respective LIWC categories) and how they positioned themselves in relation to the situation (e.g., as involved vs. detached, which were captured by a combination of LIWC categories, such as pronouns and verb tenses; Nook et al., 2017). This study thus represents a step toward examining emotions as rich and multifaceted phenomena, moving beyond reductive approaches that isolate single components.

Our research contributes to the acculturation literature by highlighting the multidimensionality and domain specificity of acculturation processes. First, we demonstrate that acquiring the host culture and maintaining the heritage culture are not opposing processes, as evidenced by the positive correlations between fitting with one culture and the other. This finding supports the conceptualization of acculturation as involving two independent dimensions, rather than a single continuum (e.g., Berry, 1992). Second, we illustrate the domain specificity of acculturation by showing that emotional endorsement and language use, while both related to cultural fit, do not correlate. This underscores that acculturation can manifest differently across various domains (e.g., Bornstein, 2017; Phinney and Flores, 2002), further enriching our understanding of how individuals navigate cultural integration.

### Limitations and future directions

Although the differences we observed in the descriptive analyses offer a glimpse into how majority Belgian people and Turkish immigrants to Belgium might make meaning of, or attend to and evaluate, their experiences, it is important to exercise caution. Our samples are not fully representative of the Belgian population and of the Turkish immigrant population, respectively, due to their limited size, and the fact that both samples were highly educated. Moreover, cultures are not stable entities that are manifested in each and every individual member to the same extent, but rather dynamic collections of ways of interacting with the environment that are subject to continuous change (Bolis and Schilbach, 2020; Markus and Kitayama, 1991). As reviewed above, however, our descriptive findings converge with prior research and theorizing about these cultural groups, suggesting that we are tapping into similar kind of population-level observations that we would get from a more representative sample. It is also the case that the presence of similarities in some demographic factors (e.g., high levels of education) can increase the comparability of samples. Therefore, with regard to the fit of Turkish migrants with majority Belgians, which is the main interest of the present study, sample considerations contextualize but do not necessarily hamper our conclusions.

In the present study, we aimed to incorporate language into the research on cultural fit in meaning-making within emotional contexts, using natural language descriptions of emotional events. However, it remains an open question whether our findings specifically pertain to emotional experiences or extend to any kind of experience in general. Before calculating cultural fit scores, we assessed the reliability of these scores across different types of situations (positive/negative and relationship−/autonomy-promoting). Results revealed poor reliability for emotional fit and acceptable reliability for language fit. This suggests that normative patterns in emotion ratings vary significantly across emotional situations, whereas normative patterns in language use are more consistent. This consistency in language use may hint at normativity in language use in a broader sense, such that the use of language is less variable across different types of experiences, whether emotional in nature or not. Since our primary interest in emotions stems from the belief that they reveal general values and worldviews, these concerns do not invalidate our findings even if the linguistic patterns captured by LIWC reflect general values and worldviews rather than being limited to the domain of emotion. Future studies could explore whether there are normative language patterns specifically associated with emotional experiences.

Another limitation of the current approach to measuring cultural fit is that the exact properties causing differences in fit are not directly observable; only overall estimates of fit are compared. Although we explored the differences between cultural groups in individual features—both of emotion endorsement and word use—the fit approach examines their relative co-occurrence. Including a wide range of features provided us with a more comprehensive assessment of fit than approaches that target similarity in single feature (e.g., Townsend et al., 2014; Tsai et al., 2004). At the same time, the specific (combinations of) features that drive (mis)fit are yet to be examined. Our interpretation of the lack of association between the two fit measures based on their relative bearings on attentional and evaluative processes also remains speculative. The lack of association between the cultural fit measures also aligns with previous observations, indicating that constructs that are linked at the group (i.e., cultural) level do not always correlate at the individual level (Na et al., 2010). We found expected cultural differences in both measures of cultural fit—although less reliable in emotional fit than in language fit. However, the extent of fitting with the culture in one domain (e.g., emotions) did not necessarily coincide with the extent of fitting in the other domain (e.g., language) within individuals.

Future research is needed to explore these possibilities further; for instance, by examining the emotion ratings and word use in combination, rather than assessing them separately. One idea would be to examine the clustering of emotion ratings and language features to reveal meaningful patterns of attention and evaluation across individuals and cultures. That is, feeling angry while focusing on social concerns vs. personal concerns (or feeling angry vs. embarrassed while focusing on social concerns) represent different types of experiencing the world, which might be more frequent in some cultures over others, although it is not possible to distinguish only relying on one feature (e.g., feeling angry, or focusing on social concerns). In a recent application of a similar method, Boiger et al. (2018) demonstrated different types of anger and shame based on the associated appraisals and action tendencies and found that the distribution of these different types across cultures systematically differ. In a similar vein, combining emotion ratings and language we can unveil culturally prevalent ways of experiencing, or conceptualizing, emotions.

## Conclusion

Taken together, the findings from the present study illustrate the valuable insights a multimethod approach may provide into the intricate and diverse nature of cultural fit of experience in emotional contexts. We assessed cultural fit based on the words people used while describing recent emotional situations and the intensity ratings they provided for these same events. We demonstrated that cultural differences can be captured by people’s language patterns to a better extent than by their emotional patterns; people have higher language fit with their own culture than the other, showing face validity for the language fit approach. Potential reasons for the inconsistencies of expected cultural differences in emotional fit were discussed. Our tests of convergent validity were not met—language fit and emotional fit were not positively related—leaving questions for future research about why these measures deviate and what they each represent. It can be argued that emotional fit and language fit revealed differences in migrants’ patterns of attention and evaluation that were not captured by one or the other alone. Future research will need to assess predictive validity by examining whether emotional fit and language fit are differentially associated with relational, psychological, and societal outcomes of interest in the context of immigration.

## Data Availability

The datasets presented in this study can be found in online repositories. The names of the repository/repositories and accession number(s) can be found in the article/Supplementary material.

## Footnotes

^1^A subset of 100 Turkish participants was selected using random number generation. Survey data were missing for one of these participants, so another participant with full data was randomly selected as a replacement. Afterwards, the survey data were located for the initial participant, bringing the final sample size to 101 participants.

^2^5 negative relationship-promoting, 1 positive autonomy-promoting, and 3 negative autonomy-promoting situations.

^3^2 positive relationship-promoting, 4 negative relationship-promoting, 3 positive autonomy-promoting, 3 negative autonomy-promoting situations.
