# Supplementary material for: Cultural fit in emotion versus language: a study of Dutch-speaking Belgians and Turkish migrants in Belgium
Source: Front Psychol. 2025 Jan 21;15:1488779. doi: 10.3389/fpsyg.2024.1488779 (PMC11790563; doi:10.3389/fpsyg.2024.1488779)
Supplement: Supplementary file 1 [file Data_Sheet_1.docx]

**Supplementary Materials**

**Emotional fit in ratings versus language: A study of the emotional experiences of**

**Dutch-speaking Belgians and Turkish migrants in Belgium**

In this document, we report further details on the measures used, additional findings on measures that were not focal for the current purposes, and alternative sets of analyses in original languages (Dutch and Turkish) and with different composition of language features for robustness check for our findings. We also provide the study materials we used in the current study in the appendix. These are a subset of the materials that were administered in the 1^st^ wave of the large-scale longitudinal study. The whole set of materials (including questionnaires administered to both cultural samples and language tests and the network questionnaires administered to Turkish migrant sample) are made available at [OSF](https://osf.io/jb74g/) (<https://osf.io/jb74g/?view_only=f9cf9a9a466e4d558d9734197dbd9758>) along with the portion of the data necessary to replicate the reported analyses and analytic code for fit score calculations.

**Methods**

**For Analyses in Original Languages**

For initial descriptive analyses and analyses for Aim 2 (*Are Emotional Fit and Language Fit Associated with Each Other?*) reported in the main text of our manuscript, we conducted extra analyses in original languages for robustness check. Analyses in original languages were not conducted for Aim 1 (*Do Belgian and Turkish participants evidenced distinct patterns of emotion endorsement and word use?*) because comparing language fit across cultures using transcripts and measurement tools (i.e., LIWC) in different languages seemed tautological.

We submitted the situation-level, participant-only documents to the Dutch (Boot et al., 2017) and Turkish (Muderrisoglu, 2012) translations of LIWC2007, respectively.

First, to ensure comparable performance of Dutch and Turkish LIWC, we checked the coverage of the two dictionaries in our transcripts. Dutch LIWC captured 91.87% of the words used in the Dutch transcripts, whereas Turkish LIWC captured 88.70%. To account for these differences in LIWC coverage, we corrected all scores by the percentage of all words captured by LIWC, by multiplying each category score with the word count, and dividing this value to the proportion of the words covered by LIWC to the total word count. For example, the dictionary corrected percentage of the category Affect was equal to:

*(Affect*WC)/(Dic/WC)*

[with Affect: Percentage of words in a transcript belonging to the category Affect, WC: word count, Dic: percentage of words covered by LIWC dictionary]

We used these corrected percentages rather than the raw ones in all the cross-linguistic analyses.

***Language fit.*** To calculate cultural fit in language *within cultural groups* (Turkish newcomers’ fit to the average Turkish pattern and Belgian majorities’ fit to the average Belgian pattern), we used the same approach we took in the main analyses of our manuscript, that is, including all the categories that exist in the respective LIWC dictionaries, except for *Punctuations.* For instance, Turkish LIWC contains categories to differentiate pronouns that are explicitly stated and that are dropped but can be inferred through verb inflections (e.g., *I*, *Verb I,* and *Total I* that represents the parent category of the former two), which Dutch or English LIWC do not capture; we retained these language-specific categories for the within-Turkish analyses. This allowed us to capture linguistic features of the given language as much as possible.

For the *between-language analyses* (Turkish newcomers’ fit to the average Belgian pattern), we prioritized comparability across languages. For example, we excluded *Articles* from Dutch LIWC because it does not exist in Turkish LIWC, *Imperative* from Turkish LIWC as it does not exist in Dutch LIWC. We also combined categories in Turkish LIWC that are not distinguished in Dutch LIWC (i.e., *Singular you* and *Plural you* with their verb inflected versions*,* and *Present tense* and *Aorist tense*).

Then, to assess the equivalence of the categories that are shared between the two languages, we compared their performance on a parallel corpus of 2,680 sentence-aligned TED2020 talks translated into Dutch and Turkish (accessed via <https://opus.nlpl.eu/TED2020.php>; Reimers & Gurevych, 2020). We calculated correlations between the Dutch and Turkish scores for each category and effect sizes for their difference. Following prior work on validating LIWC across languages (Boot et al., 2017; Dudău & Sava, 2022; Meier et al., 2019), we aimed for high correlations (*r* ≥ .50). We excluded 11 categories that fell under this threshold (.02 ≥ *r* ≥ .46).

Previous work on validation of LIWC to various languages (Dudău & Sava, 2022; Igarashi et al., 2022; Meier et al., 2019) find differences between categories across languages (up to Hedges’ *g* = 4.08 between German and English and Cohen’s *d* = 8.48 between Romanian and English) and these differences are deemed justifiable due to language specificities (Dudău & Sava, 2022). The average effect size for the categories we included in the profiles was *d* = .96 (ranging from .52 to 1.91). As these differences are conventionally tolerated in the field, we did not take any exclusion decision based on them.

Table S7 lists all LIWC2007 categories, with inclusion decision and rationale for exclusion for three types of fit calculation (within Turkish, within Dutch, and between languages).

**For analyses with different composition of language features**

For analyses for Aim 1 (*Do Belgian and Turkish participants evidenced distinct patterns of emotion endorsement and word use?*) and Aim 2 (*Are Emotional Fit and Language Fit Associated with Each Other?*) reported in the main text of our manuscript, we conducted extra analyses using an alternative set of LIWC categories in the calculation of language fit scores.

We selected categories under Psychological Processes (Pennebaker et al., 2007) that are thought to be most relevant for describing emotional experience (i.e., affective processes, social processes, cognitive processes, perceptual processes, biological processes, and their sub-categories; 25 categories in total). This allowed us not only to zoom into the “content” of emotional experience and the relative emphasis given to different aspects of it, for a more focused test of association between language fit and emotional fit in the domain of emotions (Aim 2), but also to ensure that our results are not heavily influenced by function words, some of which does not have equivalents in the original languages (e.g., *articles* do not exist in Turkish) and others are used differently across languages (e.g., pro-drop characteristic of Turkish), which might make the comparison in English questionable.

**Results and Discussion**

**Analyses in Original Languages**

***Initial Descriptive Analyses*.** Significant cultural differences in word use that are reported in the manuscript were all replicated with the transcripts in original languages (Turkish and Dutch; see Table S8). Additionally, these parallel analyses also showed that Turkish people used more positive affect (*t*(198) = 14.41, *p* < .001, 95% CI [1.926; 2.537]) and general affect words (*t*(198) = 3.64, *p* < .001, 95% CI [0.196; 0.659]) than their Belgian counterparts. These results must be treated with caution due to measurement variance problem explained in the main text.

***Aim 2.*** Associations between emotional fit and language fit were tested using language fit calculated in original languages***.***

The results were similar in that they do not offer support for a consistent relationship between two fit scores. Different from the main analyses was that, the negative association between Turkish migrants’ emotional fit and language fit with the Belgians held for negative autonomy-promoting situations, *r*(95) = -.21, *p* = .04, but not for negative relationship-promoting situations, *r*(94) = -.12, *p* = .23.

Additionally, Belgians’ emotional fit and language fit with the Belgian culture was positively associated in positive relationship-promoting situations (*r*(95) = .25, *p* = .01) and negatively associated in negative relationship-promoting situations (*r*(92) = -.24, *p* = .02). All associations with alternative language fit scores can be seen in Table S9.

**Analyses with different composition of language features**

***Aim 1***. Within- and between-culture comparisons in language fit scores were conducted using paired-sample t-tests and independent samples t-tests.

Results revealed supporting evidence for all comparisons in expected direction, except for the between-culture comparisons of language fit with the Turkish cultural pattern in positive autonomy-promoting situations (*p* = .86), such that Turkish people did not fit better to the Turkish pattern in this situation type compared to their Belgian counterparts (see Table S10 for all comparisons).

***Aim 2.*** Associations between emotional fit and language fit were tested using language fit calculated with the alternative subset of LIWC categories***.***

The results, again, were similar in that they do not offer support for a consistent relationship between two fit scores. Although the association between Turkish migrants’ emotional fit and language fit with the Belgians in negative situations were still negative they were not significant (*p*s = .10). In these analyses, negative association was only significant for

positive autonomy-promoting situations, *r*(98) = -.23, *p* = .02. All associations with alternative language fit scores can be seen in Table S9.

Although results of alternative analyses diverged from the main analyses at times, the similarities were more pronounced. First, we replicated the cross-cultural comparisons of language fit in the large majority of the analyses, except for only one situation-type in one set of analyses (i.e., between-culture comparison). The expected differences were found in all within-culture comparisons and for other three situation-types in between-culture comparisons. Second, across three different ways of calculating language fit scores, we did not find evidence for a consistent relationship between emotional fit and language fit. Moreover, across different analyses, the negative association was more pronounced in between-culture analyses (i.e., between Turkish migrants’ emotional fit and language fit with the Belgians) than within-culture analyses, suggesting that the relationship between two types of cultural fit might be operating differently between cultures than it does within cultures.

**Table S1.**

*Situational prompts from the Emotional Patterns Questionnaire (EPQ)*

| Valence | Interpersonal Motive | Prompt |
| --- | --- | --- |
| Positive | Relationship-promoting | “Recall a situation that made you feel good about yourself. For example, you may have felt proud or better than others.” |
| Positive | Autonomy-promoting | “Recall a situation that made you feel good about your relationships with others. For example, you may have felt close, respectful, or friendly towards others.” |
| Negative | Relationship-promoting | “Recall a situation that made you feel bad about something you did in a relationship with others. For example, you may have felt ashamed or guilty.” |
| Negative | Autonomy-promoting | “Recall a situation that made you feel bad about something someone else did. For example, you may have felt angry or frustrated.” |

**Table S2.**

*Mean ratings of four emotion scales per situation type*

|  | Prompted Situation | | | | | |  |  |
| --- | --- | --- | --- | --- | --- | --- | --- | --- |
| Emotion Scale | Positive  relationship- promoting | | Positive  autonomy- promoting | Negative  relationship- promoting | | Negative  autonomy- promoting |  |  |
| *Turkish migrants* |  | |  |  | |  |  |  |
| Positive relationship-promoting | | **3.96*** | 3.76 | | 2.33 | 2.47 | | |
| Positive autonomy-promoting | | 3.78 | **4.16*** | | 1.50 | 1.58 | | |
| Negative relationship-promoting | | 1.58 | 1.01 | | **3.10*** | 2.02 | | |
| Negative autonomy-promoting | | 1.26 | 1.35 | | 2.76 | **3.98*** | | |
| *Belgians* |  | |  |  | |  |  |  |
| Positive relationship-promoting | | **4.08*** | 3.62 | | 1.78 | 1.79 | | |
| Positive autonomy-promoting | | 3.69 | **4.02*** | | 1.10 | 1.10 | | |
| Negative relationship-promoting | | 1.32 | 1.25 | | **3.36*** | 1.83 | | |
| Negative autonomy-promoting | | 1.13 | 1.26 | | 2.76 | **3.99*** | | |

*Note.* The emotions included in each scale are: positive relationship-promoting (*respectful, close, helpful*); positive autonomy-promoting (*happy, elated, proud*); negative relationship-promoting (*ashamed, guilty, indebted*); negative autonomy-promoting (*angry, frustrated, resentful*). Emotion scales with the highest mean rating per situation type are represented in bold. **p* < .05.

**Table S3.**

*LIWC2007 word categories included in the main analyses (taken from Pennebaker et al., 2007)*

| **Category** | **Example** | **Words in category** |
| --- | --- | --- |
| *Linguistic Processes* |  |  |
| Total function words |  | 464 |
| Total pronouns | I, them, itself | 116 |
| Personal pronouns | I, them, her | 70 |
| 1^st^ person singular | I, me, mine | 12 |
| 1^st^ person plural | We, us, our | 12 |
| 2^nd^ person singular | You, your, thou | 20 |
| 3^rd^ person singular | She, her, him | 17 |
| 3^rd^ person plural | They, their, they’d | 10 |
| Impersonal pronouns | It, it’s, those | 46 |
| Articles | A, an, the | 3 |
| Verbs | Walk, went, see | 383 |
| Auxiliary verbs | Am, will, have | 144 |
| Past tense | Went, ran, had | 145 |
| Present tense | Is, does, hear | 169 |
| Future tense | Will, gonna | 48 |
| Adverbs | Very, really, quickly | 69 |
| Prepositions | To, with, above | 60 |
| Conjunctions | And, but, whereas | 28 |
| Negations | No, not, never | 57 |
| Quantifiers | Few, many, much | 89 |
| Numbers | Second, thousand | 34 |
| Swear words | Damn, piss, fuck | 53 |
| *Psychological Processes* |  |  |
| Social processes | Mate, talk, they, child | 455 |
| Family | Daughter, husband, aunt | 64 |
| Friends | Buddy, friend, neighbor | 37 |
| Humans | Adult, baby, boy | 61 |
| Affective processes | Happy, cried, abandon | 915 |
| Positive emotion | Love, nice, sweet | 406 |
| Negative emotion | Hurt, ugly, nasty | 499 |
| Anxiety | Worried, fearful, nervous | 91 |
| Anger | Hate, kill, annoyed | 184 |
| Sadness | Crying, grief, sad | 101 |
| Cognitive processes | Cause, know, ought | 730 |
| Insight | Think, know, consider | 195 |
| Causation | Because, effect, hence | 108 |
| Discrepancy | Should, would, could | 76 |
| Tentative | Maybe, perhaps, guess | 155 |
| Certainty | Always, never | 83 |
| Inhibition | Block, constrain, stop | 111 |
| Inclusion | And, with, include | 18 |
| Exclusion | But, without, exclude | 17 |
| Perceptual processes | Observing, heard, feeling | 273 |
| See | View, saw, seen | 72 |
| Hear | Listen, hearing | 51 |
| Feel | Feels, touch | 75 |
| Biological processes | Eat, blood, pain | 567 |
| Body | Cheek, hands, spit | 180 |
| Health | Clinic, flu, pill | 236 |
| Sexual | Horny, love, incest | 96 |
| Ingestion | Dish, eat, pizza | 111 |
| Relativity | Area, bend, exit, stop | 638 |
| Motion | Arrive, car, go | 168 |
| Space | Down, in, thin | 220 |
| Time | End, until, season | 239 |
| *Personal concerns* |  |  |
| Work | Job, majors, xerox | 327 |
| Achievement | Earn, hero, win | 186 |
| Leisure | Cook, chat, movie | 229 |
| Home | Apartment, kitchen, family | 93 |
| Money | Audit, cash, owe | 173 |
| Religion | Altar, church, mosque | 159 |
| Death | Bury, coffin, kill | 62 |
| *Spoken categories* |  |  |
| Assent | Agree, OK, yes | 30 |
| Nonfluencies | Er, hm, umm | 8 |
| Filler | blah, Imean, youknow | 9 |

**Table S4.**

*Comparison of emotion ratings between cultural samples*

|  | *Mean (SD)* | | | *T-test for*  *Equality of Means* | | *95% Confidence Interval of the Mean Difference* | |
| --- | --- | --- | --- | --- | --- | --- | --- |
| *Emotions* | | *Turkish* | *Belgian* | *t (df)* | *Mean difference (SE)* | Lower | Upper |
| Proud | | 3.71 (.66) | 2.40 (.55) | 3.539*** (192) | 0.31 (.09) | 0.138 | 0.484 |
| Guilty | | 1.98 (.58) | 2.04 (.51) | -.702 (191) | -0.06 (.08) | -0.210 | 0.100 |
| Respectful | | 3.62 (.68) | 3 (.70) | 6.274*** (192) | 0.63 (.10) | 0.429 | 0.822 |
| Angry | | 2.47 (.61) | 2.20 (.53) | 3.272*** (190) | 0.27 (.08) | 0.108 | 0.435 |
| Happy | | 2.94 (.36) | 2.59 (.47) | 5.830*** (193) | 0.35 (.06) | 0.232 | 0.469 |
| Resentful | | 2.18 (.61) | 2.15 (.60) | .358 (192) | 0.03 (.09) | -0.140 | 0.202 |
| Close | | 2.97 (.61) | 2.83 (.66) | 1.468 (193) | 0.13 (.09) | -0.046 | 0.312 |
| Ashamed | | 2.13 (.56) | 1.93 (.53) | 2.569** (192) | 0.20 (.08) | 0.047 | 0.357 |
| Elated | | 2.68 (.64) | 2.42 (.53) | 3.089** (193) | 0.26 (.10) | 0.095 | 0.429 |
| Helpful | | 2.82 (.78) | 2.60 (.66) | 2.090* (191) | 0.22 (.10) | 0.012 | 0.424 |
| Indebted | | 1.85 (.72) | 1.87 (.71) | -.207 (191) | -0.02 (.10) | -0.225 | 0.182 |
| Worried | | 2.59 (.71) | 2.45 (.81) | 1.261 (192) | 0.14 (.11) | -0.078 | 0.354 |
| Nervous | | 2.74 (.69) | 2.23 (.80) | 4.767*** (192) | 0.51 (.11) | 0.300 | 0.723 |
| Fearful | | 1.75 (.65) | 1.63 (.67) | 1.164 (191) | 0.11 (.10) | -0.077 | 0.298 |
| Sad | | 2.52 (.63) | 2.02 (.70) | 5.210*** (190) | 0.50 (.10) | 0.312 | 0.691 |

*Note.* All analyses were conducted with 2-tailed testing. **p* ≤ .05, ***p* ≤ .01, ****p* ≤ .001.

**Table S5.**

*Comparison of word use between cultural samples*

|  | | *Mean (SD)* | | | | *T-test for*  *Equality of Means* | | *95% Confidence Interval of the Mean Difference* | |
| --- | --- | --- | --- | --- | --- | --- | --- | --- | --- |
| LIWC categories | *Turkish* | | | *Belgian* | *t* | | *Mean difference (SE)* | *Lower* | *Upper* |
| Function words | | | 65.75 (1.40) | 67.03 (2.54) | -4.40*** | | -1.28 (.29) | -1.847 | -.703 |
| Pronouns | | | 23.82 (1.78) | 22.01 (1.95) | 6.84*** | | 1.81 (.26) | 1.285 | 2.326 |
| Personal Pronouns | | | 14.77 (1.68) | 11.54 (1.58) | 13.97*** | | 3.22 (.23) | 2.767 | 3.677 |
| I | | | 8.77 (1.79) | 6.42 (1.59) | 9.79*** | | 2.35 (.24) | 1.875 | 2.820 |
| We | | | 1.54 (0.93) | 0.85 (0.47) | 6.60*** | | .69 (.10) | .482 | .893 |
| You | | | 1.52 (0.77) | 1.93 (0.96) | -3.41*** | | -.42 (.12) | -.659 | -.176 |
| She/he | | | 1.93 (0.90) | 1.51 (0.76) | 3.54*** | | .42 (.12) | .185 | .648 |
| They | | | 1.013 (0.57) | 0.83 (0.48) | 2.51** | | .19 (.07) | .040 | .335 |
| Impersonal Pronouns | | | 9.05 (1.13) | 10.47 (1.42) | -7.81*** | | -1.42 (.18) | -1.775 | -1.059 |
| Articles | | | 5.96 (0.85) | 5.20 (0.86) | 6.29*** | | .76 (.12) | .520 | .995 |
| Verbs | | | 18.77 (1.33) | 16.74 (1.27) | 11.02*** | | 2.03 (.18) | 1.668 | 2.395 |
| Auxiliary Verbs | | | 11.13 (1.02) | 10.96 (0.98) | 1.22 | | 0.17 (.14) | -0.107 | 0.451 |
| Past Tense | | | 7.38 (2.05) | 5.78 (1.84) | 5.82*** | | 1.60 (.28) | 1.059 | 2.145 |
| Present Tense | | | 9.40 (2.04) | 9.45 (2.06) | -0.18 | | -0.05 (.29) | -0.624 | 0.518 |
| Future Tense | | | 0.92 (0.30) | 0.75 (0.24) | 4.46*** | | 0.17 (.04) | 0.095 | 0.246 |
| Adverbs | | | 6.71 (0.87) | 8.77 (1.07) | -14.94*** | | -2.06 (.14) | -2.330 | -1.787 |
| Prepositions | | | 10.62 (0.93) | 9.88 (1.19) | 4.84*** | | 0.73 (.15) | 0.435 | 1.032 |
| Conjunctions | | | 6.85 (0.80) | 10.52 (1.19) | -25.64*** | | -3.67 (.14) | -3.956 | -3.391 |
| Negations | | | 2.80 (0.53) | 2.95 (0.83) | -1.52 | | -0.15 (.10) | -0.345 | 0.045 |
| Quantifiers | | | 2.83 (0.55) | 2.59 (0.46) | 3.28*** | | 0.24 (.07) | 0.094 | 0.378 |
| Numbers | | | 1.26 (0.42) | 1.29 (1.48) | -0.15 | | -0.02 (.15) | -0.326 | 0.279 |
| Swear Words | | | 0.01 (0.02) | 0.03 (0.05) | -3.56*** | | -0.02 (.00) | -0.028 | -0.008 |
| Social Processes | | | 11.90 (2.32) | 10.22 (1.63) | 5.92*** | | 1.68 (.28) | 1.119 | 2.237 |
| Family | | | 0.27 (0.28) | 0.41 (0.35) | -3.15** | | -0.14 (.04) | -0.229 | -0.053 |
| Friend | | | 0.39 (0.27) | 0.33 (0.20) | 1.70 | | 0.06 (.03) | -0.009 | 0.125 |
| Humans | | | 1.05 (0.46) | 0.66 (0.36) | 6.56*** | | 0.38 (.06) | 0.269 | 0.500 |
| Affective Processes | | | 5.21 (0.91) | 5.04 (0.81) | 1.37 | | 0.17 (.12) | -0.074 | 0.407 |
| Positive Emotion | | | 3.41 (0.81) | 3.40 (0.64) | 0.10 | | 0.01 (.10) | -0.193 | 0.213 |
| Negative Emotion | | | 1.75 (0.44) | 1.60 (0.45) | 2.45* | | 0.15 (.06) | 0.030 | 0.279 |
| Anxiety | | | 0.39 (0.19) | 0.31 (0.17) | 2.92** | | 0.08 (.03) | 0.024 | 0.126 |
| Anger | | | 0.33 (0.18) | 0.41 (0.21) | -3.05** | | -0.08 (.03) | -0.140 | -0.030 |
| Sadness | | | 0.31 (0.17) | 0.28 (0.19) | 1.23 | | 0.03 (.03) | -0.019 | 0.081 |
| Cognitive Processes | | | 18.66 (1.53) | 19.84 (1.55) | -5.40*** | | -1.18 (.22) | -1.606 | -0.747 |
| Insight | | | 4.01 (0.91) | 3.09 (0.66) | 8.21*** | | 0.92 (.11) | 0.702 | 1.145 |
| Causality | | | 2.37 (0.51) | 1.97 (0.43) | 6.03*** | | 0.40 (.07) | 0.270 | 0.533 |
| Discrepancy | | | 2.09 (0.50) | 1.59 (0.35) | 8.19*** | | 0.50 (.06) | 0.382 | 0.624 |
| Tentative | | | 3.01 (0.66) | 3.22 (0.90) | -1.84 | | -0.21 (.11) | -0.426 | 0.015 |
| Certainty | | | 1.08 (0.28) | 1.45 (0.37) | -8.23*** | | -0.38 (.05) | -0.469 | -0.288 |
| Inhibition | | | 0.29 (0.15) | 0.30 (0.14) | -0.59 | | -0.01 (.02) | -0.052 | 0.028 |
| Inclusion | | | 4.15 (0.89) | 5.31 (0.91) | -9.09*** | | -1.16 (.13) | -1.407 | -0.905 |
| Exclusion | | | 3.42 (0.69) | 4.77 (1.09) | -10.49*** | | -1.35 (.13) | -1.608 | -1.099 |
| Perceptual Processes | | | 2.87 (0.71) | 2.60 (0.52) | 3.07** | | 0.27 (.09) | 0.096 | 0.443 |
| See | | | 0.52 (0.22) | 0.51 (0.20) | 0.19 | | 0.01 (.03) | -0.053 | 0.064 |
| Hear | | | 1.27 (0.45) | 1.13 (0.37) | 2.41* | | 0.14 (.06) | 0.025 | 0.255 |
| Feel | | | 0.95 (0.42) | 0.90 (0.32) | 0.92 | | 0.05 (.05) | -0.056 | 0.153 |
| Biological Processes | | | 0.80 (0.34) | 0.74 (0.36) | 1.18 | | 0.06 (.05) | -0.039 | 0.155 |
| Body | | | 0.25 (0.14) | 0.29 (0.17) | -1.75 | | -0.04 (.02) | -0.083 | 0.005 |
| Health | | | 0.31 (0.20) | 0.28 (0.21) | 0.95 | | 0.03 (.03) | -0.029 | 0.084 |
| Sexual | | | 0.06 (0.07) | 0.04 (0.06) | 1.57 | | 0.01 (.01) | -0.004 | 0.033 |
| Ingestion | | | 0.19 (0.21) | 0.14 (0.14) | 2.21* | | 0.06 (.02) | 0.006 | 0.104 |
| Relativity | | | 12.27 (1.43) | 12.33 (1.33) | -0.31 | | -0.06 (.19) | -0.445 | 0.324 |
| Motion | | | 1.74 (0.46) | 1.76 (0.40) | -0.29 | | -0.02 (.06) | -0.137 | 0.102 |
| Space | | | 5.11 (0.68) | 4.39 (0.71) | 7.32*** | | 0.72 (.10) | 0.527 | 0.915 |
| Time | | | 5.03 (0.90) | 5.93 (0.92) | -7.02*** | | -0.90 (.13) | -1.153 | -0.647 |
| Work | | | 2.08 (1.016) | 1.43 (0.67) | 5.39*** | | 0.66 (.12) | 0.416 | 0.896 |
| Achieve | | | 1.73 (0.57) | 1.42 (0.36) | 4.69*** | | 0.32 (.07) | 0.183 | 0.448 |
| Leisure | | | 0.46 (0.26) | 0.51 (0.31) | -1.26 | | -0.05 (.04) | -0.132 | 0.029 |
| Home | | | 0.43 (0.34) | 0.40 (0.22) | 0.50 | | 0.02 (.04) | -0.060 | 0.101 |
| Money | | | 0.41 (0.35) | 0.22 (0.20) | 4.75*** | | 0.19 (.04) | 0.112 | 0.270 |
| Religion | | | 0.07 (0.10) | 0.03 (0.04) | 3.64*** | | 0.04 (.01) | 0.018 | 0.062 |
| Death | | | 0.02 (0.04) | 0.02 (0.04) | -0.65 | | 0.00 (.01) | -0.014 | 0.007 |
| Assent | | | 0.47 (0.26) | 3.25 (1.21) | -22.37*** | | -2.78 (.12) | -3.022 | -2.532 |
| Nonfluencies | | | 0.29 (0.54) | 0.58 (0.52) | -3.91*** | | -0.29 (.08) | -0.441 | -0.145 |
| Filler | | | 0.77 (0.33) | 0.57 (0.31) | 4.34*** | | 0.20 (.05) | 0.108 | 0.288 |

*Note.* df = 198 for all comparisons. All analyses were conducted with 2-tailed testing. **p* ≤ .05, ***p* ≤ .01, ****p* ≤ .001.

**Table S6.**

*Comparison of correlations between fit with one culture and with the other*

| **Situation Type** | ***r_rating*** | ***r_language*** | ***95% CI for r difference*** |
| --- | --- | --- | --- |
| *Turkish* |  |  |  |
| Positive relationship-promoting | .99 | .71 | [.209, .401] |
| Positive autonomy-promoting | .99 | .80 | [.136, .288] |
| Negative relationship-promoting | .86 | .70 | [.036, .261] |
| Negative autonomy-promoting | .90 | .65 | [.137, .387] |
| *Belgian* |  |  |  |
| Positive relationship-promoting | .98 | .71 | [.175, .373] |
| Positive autonomy-promoting | .99 | .70 | [.201, .408] |
| Negative relationship-promoting | .76 | .74 | [-.107, .150] |
| Negative autonomy-promoting | .97 | .69 | [.185, .405] |

*Note.* r_rating represents the correlation between rating fit with Belgian culture and rating fit with Turkish culture across cultural groups and situation types. r_language represents the correlation between language fit with Belgian culture and language fit with Turkish culture across cultural groups and situation types. 95% CI shows the difference between correlation coefficients for rating fit and language fit (Diedenhofen & Musch, 2015; Zou, 2007).

**Table S7**

*Word categories included and the reason for exclusion across set of analyses*

|  | Within-language,  Turkish | Within-language,  Dutch | Between-language |
| --- | --- | --- | --- |
| Word category | Included | Included | Included  (exclusion reason) |
| Function words | Y | Y | Y |
| Pronouns | Y | Y | Y |
| Personal pronouns | Y | Y | Y |
| I | Y | Y | Y  [For Turkish:  Total I] |
| Verb I | Y | NA |  |
| Total I | Y | NA |  |
| We | Y | Y | Y  [For Turkish:  Total we] |
| Verb we | Y | NA |  |
| Total we | Y | NA |  |
| You | NA | Y | Y  [For Turkish: Total you singular + Total you plural] |
| You singular | Y | NA |  |
| Verb you singular | Y | NA |  |
| Total you singular | Y | NA |  |
| You plural | Y | NA |  |
| Verb you plural | Y | NA |  |
| Total you plural | Y | NA |  |
| She/he | Y | Y | Y  [For Turkish:  Total she/he] |
| Verb she/he | Y | NA |  |
| Total she/he | Y | NA |  |
| They | Y | Y | Y  [For Turkish:  Total they] |
| Verb they | Y | NA |  |
| Total they | Y | NA |  |
| Impersonal pronouns | Y | Y | Y |
| Articles | NA | Y | N (NA in Turkish) |
| Verbs | Y | Y | Y |
| Auxiliary verbs | NA | Y | N (NA in Turkish) |
| Passive verbs | Y | NA | N (NA in Dutch) |
| Past tense | Y | Y | Y |
| Past simple (“-di”) | Y | NA | N (NA in Dutch) |
| Past perfect (“-miş”) | Y | NA | N (NA in Dutch) |
| Present tense | Y | Y | Y [For Turkish:  Present + Aorist] |
| Aorist tense | Y | NA |  |
| Future tense | Y | Y | N (not equivalent) |
| Total “-miş” | Y | NA | N (NA in Dutch) |
| Modalities | Y | NA | N (NA in Dutch) |
| Must total | Y | NA | N (NA in Dutch) |
| Must Past | Y | NA | N (NA in Dutch) |
| Can total | Y | NA | N (NA in Dutch) |
| Can past | Y | NA | N (NA in Dutch) |
| Desire total | Y | NA | N (NA in Dutch) |
| Desire past | Y | NA | N (NA in Dutch) |
| Imperative | Y | NA | N (NA in Dutch) |
| Descriptive | Y | NA | N (NA in Dutch) |
| Adverbs | NA | Y | N (NA in Turkish) |
| Prepositions | Y | Y | N (not equivalent) |
| Conjugations | Y | Y | N (not equivalent) |
| Negations | Y | Y | Y |
| Quantifiers | Y | Y | Y |
| Numbers | Y | Y | N (not equivalent) |
| Questions | Y | NA | N (NA in Dutch) |
| Swear words | Y | Y | N (not equivalent) |
| Social processes | Y | Y | Y |
| Family | Y | Y | Y |
| Friends | Y | Y | Y |
| Humans | Y | Y | Y |
| Affective processes | Y | Y | Y |
| Positive emotion | Y | Y | Y |
| Negative emotion | Y | Y | Y |
| Anxiety | Y | Y | Y |
| Anger | Y | Y | Y |
| Sadness | Y | Y | Y |
| Cognitive processes | Y | Y | Y |
| Insight | Y | Y | Y |
| Causation | Y | Y | N (not equivalent) |
| Discrepancy | Y | Y | Y |
| Tentative | Y | Y | Y |
| Certainty | Y | Y | Y |
| Inhibition | Y | Y | N (not equivalent) |
| Inclusive | Y | Y | N (not equivalent) |
| Exclusive | Y | Y | Y |
| Perceptual processes | Y | Y | Y |
| See | Y | Y | Y |
| Hear | Y | Y | Y |
| Feel | Y | Y | N (not equivalent) |
| Biological processes | Y | Y | Y |
| Body | Y | Y | Y |
| Health | Y | Y | Y |
| Sexual | Y | Y | Y |
| Ingestion | Y | Y | Y |
| Relativity | Y | Y | Y |
| Motion | Y | Y | Y |
| Space | Y | Y | Y |
| Time | Y | Y | Y |
| Work | Y | Y | Y |
| Achievement | Y | Y | Y |
| Leisure | Y | Y | Y |
| Home | Y | Y | Y |
| Money | Y | Y | Y |
| Religion | Y | Y | Y |
| Death | Y | Y | Y |
| Assent | Y | Y | Y |
| Filler | Y | Y | N (not equivalent) |
| Nonfluencies | Y | Y | N (not equivalent) |

*Note.* Y: included, N: excluded. NA is used when a category does not exist in the LIWC dictionary for that language.

**Table S8.**

*Comparison of word use between cultural samples (in original languages)*

|  | *Mean (SD)* | | *T-test for*  *Equality of Means* | | *95% Confidence Interval of the Mean Difference* | |
| --- | --- | --- | --- | --- | --- | --- |
| LIWC categories | *Turkish* | *Belgian* | *t* | *Mean difference (SE)* | *Lower* | *Upper* |
| Affective Processes | 7.50 (1.24) | 5.27 (.93) | 14.41*** | 2.23 (.15) | 1.926 | 2.537 |
| Positive Emotion | 3.77 (.93) | 3.34 (.71) | 3.64*** | .43 (.12) | .196 | .659 |
| Negative Emotion | 3.14 (1.48) | 1.48 (.41) | 17.13*** | 1.66 (.10) | 1.472 | 1.855 |
| Social Processes | 10.33 (2) | 9.81 (1.51) | 2.06* | .52 (.25) | .022 | 1.010 |
| She/he (only pronoun in Turkish) | 3.20 (.99) | .97 (.50) | 20.64*** | 2.23 (.11) | 2.015 | 2.441 |
| She/he (Total she/he in Turkish) | 8.37 (1.42) |  | 49*** | 7.40 (.15) | 7.10 | 7.695 |
| You (only pronoun in Turkish) | .52 (.35) | 2 (1.01) | -13.87*** | -1.48 (.11) | -1.694 | -1.272 |
| You (Total you in Turkish) | 1.09 (0.69) |  | -7.44*** | -.91 (.12) | -1.152 | -.669 |
| Achievement | 2.71 (.89) | 1.21 (.31) | 15.96*** | 1.51 (.09) | 1.319 | 1.691 |
| Certainty | 2.81 (.91) | 5.30 (1.45) | -14.60*** | -2.49 (.17) | -2.830 | -2.156 |

*Note*. *df* = 198 for all comparisons.

**Table S9.**

*Correlations between language fit (with alternative calculations) and emotional fit*

|  |  | | **In original languages** | | **With alternative set of LIWC categories** | |
| --- | --- | --- | --- | --- | --- | --- |
| **Situation Type** | ***df*** | | ***r*** | ***p*** | ***r*** | ***p*** |
| *Within-culture: Belgian majority* | |  |  |  |  |  |
| Positive relationship-promoting | 95 | | **.25** | .01 | .17 | .09 |
| Positive autonomy-promoting | 95 | | .10 | .34 | .08 | .46 |
| Negative relationship-promoting | 92 | | **-.24** | .02 | -.04 | .72 |
| Negative autonomy-promoting | 94 | | .05 | .63 | .13 | .21 |
| *Within-culture: Turkish migrants* |  | |  |  |  |  |
| Positive relationship-promoting | 99 | | -.01 | .91 | .09 | .36 |
| Positive autonomy-promoting | 98 | | .11 | .28 | -.13 | .21 |
| Negative relationship-promoting | 94 | | -.07 | .52 | -.16 | .11 |
| Negative autonomy-promoting | 95 | | -.10 | .33 | -.04 | .73 |
| *Between-culture: Turkish migrants’ fit with Belgians* | | | | | | |
| Positive relationship-promoting | 99 | | -.14 | .17 | -.06 | .53 |
| Positive autonomy-promoting | 98 | | .04 | .69 | **-.23** | .02 |
| Negative relationship-promoting | 94 | | -.12 | .23 | -.17 | .10 |
| Negative autonomy-promoting | 95 | | **-.21** | .04 | -.17 | .10 |

*Note.* The analyses in the original languages were conducted with language fit scores calculated using a) Dutch LIWC categories for analyses within Belgian majority sample, b) Turkish LIWC categories for analyses within Turkish migrant sample, and c) cross-culturally comparable categories (see above for detailed explanation, and Table S8 for the list of categories used in each set of analyses). The analyses with the alternative set of LIWC categories were conducted with language fit scores calculated using a smaller set of LIWC categories, including *Social Processes, Family, Friend, Human,* *Affective Processes*, *Positive Emotion, Negative Emotion, Cognitive Processes, Insight, Causation, Discrepancy, Tentative, Certainty, Inhibition, Inclusive, Exclusive, Perceptual Processes, See, Hear, Feel, Biological Processes, Body, Health, Sexual,* and *Ingestion*. The associations that are statistically significant (*p* < .05) are represented in bold.

**Table S10.**

*Within- and between-culture comparisons in language fit*

| ***Within culture comparisons*** | | | | | | | |
| --- | --- | --- | --- | --- | --- | --- | --- |
|  |  | *Mean (SD)* | |  |  |  |  |
|  |  | *Fit to min* | *Fit to maj* | *t* | *df* | *p (two-sided)* | *correlation* |
| ***EP*** | *Turkish* | 2.28 (.35) | 2.09 (.31) | 6.99 | 100 | < .001 | .65 |
|  | *Belgian* | 2.15 (.27) | 2.39 (.32) | 8.35 | 96 | < .001 | .58 |
| ***EN*** | *Turkish* | 2.28 (.33) | 2.09 (.30) | 8.34 | 95 | < .001 | .75 |
|  | *Belgian* | 2.19 (.28) | 2.45 (.29) | 8.81 | 94 | < .001 | .53 |
| ***DP*** | *Turkish* | 2.14 (.35) | 2.01 (.29) | 7.10 | 99 | < .001 | .84 |
|  | *Belgian* | 2.15 (.31) | 2.31 (.34) | 7.61 | 96 | < .001 | .80 |
| ***DN*** | *Turkish* | 2.37 (.36) | 2.12 (.31) | 9.40 | 96 | < .001 | .70 |
|  | *Belgian* | 2.20 (.33) | 2.49 (.31) | 8.69 | 95 | < .001 | .50 |
| ***Between culture comparisons*** | | | | | | | |
|  |  | *Turkish - Belgian (SE)* | | *t* | *df* | *p (two-sided)* |  |
| ***EP*** | *Fit to Turkish* | .13 (.04) | | 2.90 | 195 | .004 |  |
|  | *Fit to Belgian* | -.30 (.04) | | -6.60 | 195 | < .001 |  |
| ***EN*** | *Fit to Turkish* | .09 (.04) | | 1.97 | 189 | .05 |  |
|  | *Fit to Belgian* | -.35 (.04) | | -8.28 | 189 | < .001 |  |
| ***DP*** | *Fit to Turkish* | -.01 (.05) | | -.180 | 195 | .86 |  |
|  | *Fit to Belgian* | -.30 (.04) | | -6.73 | 195 | < .001 |  |
| ***DN*** | *Fit to Turkish* | .17 (.05) | | 3.42 | 191 | < .001 |  |
|  | *Fit to Belgian* | -.36 (.04) | | -8.18 | 191 | < .001 |  |

*Note.* These analyses were conducted with language fit score calculated using a smaller set of LIWC categories, including *Social Processes, Family, Friend, Human,* *Affective Processes, Positive Emotion, Negative Emotion, Cognitive Processes, Insight, Causation, Discrepancy, Tentative, Certainty, Inhibition, Inclusive, Exclusive, Perceptual Processes, See, Hear, Feel, Biological Processes, Body, Health, Sexual,* and *Ingestion*.

**Figure S1**

*Cultural fit using profile correlations*

**
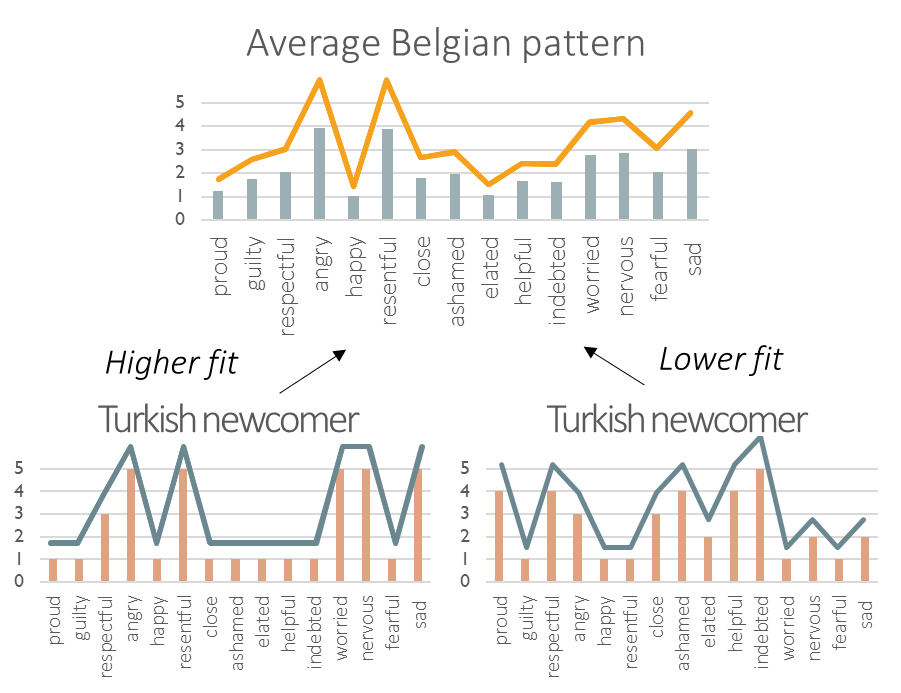
**

*Note.* This figure demonstrates profile similarity approach to cultural fit. First, we create profiles of the relevant features for each participant (e.g., two individual profiles consisting of 15 emotion intensity ratings collected from two Turkish participants are represented at the bottom). Then, we calculate the average pattern of relevant features obtained from the cultural group by averaging all the participants’ profiles in the respective cultural sample (e.g., majority Belgian average pattern of 15 emotion intensity ratings is represented at the top). Lastly, we correlate each participant’s profile with the average cultural pattern. The resulting Pearson correlation coefficients are fisher-transformed to be used as estimates of *emotional fit*, representing the similarity of an individual’s emotional pattern to the average emotional pattern in that culture within the given situation type (e.g., the patterns at the bottom left and bottom right illustrate people with a high and low fit with the majority Belgian pattern respectively).

**Appendix: Materials**

**Emotional Patterns Questionnaire (EPQ)**

First, we will ask you to describe four situations that you have experienced in the past 6 months. Some of these situations have made you feel good, others made you feel bad.

**Situation 1 (Positive relationship-promoting):** Now, we will ask you to recall one situation that made you feel good about your relationships with others. For example, you may have felt close, respectful or friendly toward the others.

**Situation 2 (Negative relationship-promoting):** Now we will ask you to recall one situation that made you feel bad about what you did in your relationship with another. For example, you may have felt ashamed or guilty.

**Situation 3 (Positive autonomy-promoting):** Now we will ask you to recall one situation that made you feel good about yourself or your behavior. For example, you may have felt proud or better than others.

**Situation 4 (Negative autonomy-promoting):** Now we will ask you to recall one situation that made you feel bad about what someone else did. For example, you may have felt angry or frustrated.

Please briefly describe what happened: (open-ended)

How strongly did you feel each of these emotions during this situation?

1-------------------------2-------------------------3-------------------------4-------------------------5

Not at all A little Somewhat Strongly Very strongly

1. Good
2. Bad
3. Proud
4. Guilty
5. Frustrated
6. Respectful towards the others
7. Angry
8. Happy
9. Resentful
10. Close to the others
11. Ashamed
12. Elated (Excited)
13. Helpful towards the others
14. Indebted
15. Calm
16. Worried
17. Nervous
18. Fearful
19. Sad
20. Surprised

**Interview Scheme**

(If the participant has difficulty remembering the situation, the interviewer may rely on the notes taken during the questionnaire and shortly

remind the participants of the situation. For example: Earlier, you described the situation in the train station where you met your friend… etc.,)

Can you give a little more detail about what happened and how you felt?

___________________________________________________________________________

I would like to ask you a few more questions about this situation. Is there a problem with that?

*(First) Questions about the situation:*

• *(If this was an interaction)* Who were you interacting with?

• Where were you?

• When did this happen? What time of day was it?

• How important was this event to you?

• How good/bad was this event to you?

*(Second) Questions about emotions*

• What did you do or what did you say or what to say or

did you want to do?

• How did you feel ?

• Have you had any sensations in your body?

• Can you tell me why you feel or act this way? What can you say about the event that caused you to feel or behave this way?

• *(If this was an interaction)* What did the other person(s) say or do?

• *(If this was an interaction)* Did this change your initial feelings or actions?

• Were there other people when this happened? Who were these people and how did they react?

**References**

Boot, P., Zijlstra, H., & Geenen, R. (2017). The Dutch translation of the linguistic inquiry and word count (LIWC) 2007 dictionary. *Dutch Journal of Applied Linguistics*, *6*(1), 65-76.

Dudău, D. P., & Sava, F. A. (2022). The development and validation of the Romanian version of Linguistic Inquiry and Word Count 2015 (Ro-LIWC2015). *Current Psychology*, *41*(6), 3597-3614.

Igarashi, T., Okuda, S., & Sasahara, K. (2022). Development of the japanese version of the linguistic inquiry and word count dictionary 2015. *Frontiers in psychology*, *13*, 841534.

Meier, T., Boyd, R. L., Pennebaker, J. W., Mehl, M. R., Martin, M., Wolf, M., & Horn, A. B. (2019). “LIWC auf Deutsch”: The development, psychometrics, and introduction of DE-LIWC2015. *PsyArXiv*, (a).

Müderrisoğlu, S. (2012). Türkçe psikolojik metin analizi programı: LIWC Türkçe. 17. *Ulusal Psikoloji Kongresi. Boğaziçi Üniversitesi, İstanbul*.

Pennebaker, J., Chung, C., Ireland, M., Gonzales, A., & Booth, R. (2007). The development and psychometric properties of LIWC2007. Austin, TX: LIWC. *URL: www. liwc. net*.

Reimers, N., & Gurevych, I. (2020). Making monolingual sentence embeddings multilingual using knowledge distillation. *arXiv preprint arXiv:2004.09813*.
